# Associations Between Keratoconus and the Level of Sex Hormones: A Cross-Sectional Study

**DOI:** 10.3389/fmed.2022.828233

**Published:** 2022-02-24

**Authors:** Xiaorui Zhao, Yifei Yuan, Tong Sun, Yu Zhang, Yueguo Chen

**Affiliations:** ^1^Department of Ophthalmology, Peking University Third Hospital, Beijing, China; ^2^Beijing Key Laboratory of Restoration of Damaged Ocular Nerve, Peking University Third Hospital, Beijing, China

**Keywords:** keratoconus, sex hormone, etiopathogenesis, proinflammatory cytokine, matrix metalloproteinases

## Abstract

**Purpose:**

To analyze the level of sex hormone in relation to keratoconus (KC).

**Methods:**

Sixty-two eyes of 62 patients (12 females, 50 males) classified as KC and 120 eyes of 120 patients (21 females, 99 males) with mild to moderate myopia and astigmatism were analyzed. Plasma samples were collected and analyzed using a chemiluminescence immunoassay to determine the concentrations of estriol (E_3_), estradiol (E_2_), progesterone (P), and testosterone (T). Corneal morphological parameters, such as the central corneal thickness (CCT), thinnest corneal thickness (TCT), and maximum simulated keratometry (K_max_), were measured using Pentacam and Sirius.

**Results:**

The mean age was 23.73 ± 5.16 years for patients with KC and 23.68 ± 6.10 years for patients treated with laser vision correction (LVC). Among the patients with KC, 12 were female (19.35%) and 50 were male (80.65%). The majority of patients with KC were between 20 and 30 years old. In female patients, the concentration of T in the KC group was significantly lower than that in the LVC group (0.86 ± 0.33 vs. 1.18 ± 0.58 nmol/L; *P* = 0.044). There were positive correlations between T, CCT (*r* = 0.395, *P* = 0.023) and TCT (*r* = 0.378, *P* = 0.030) in female patients. In male patients, E_2_ was higher in the KC group than the LVC group (143.75 ± 34.82 vs. 124.80 ± 43.56 pmol/L; *P* = 0.013), while T was significantly lower (11.59 ± 2.85 vs. 13.58 ± 4.77 nmol/L; *P* = 0.026). A positive correlation was found between E_2_ and K_max_ (*r* = 0.222, *P* = 0.007) in male patients.

**Conclusions:**

Conclusively, our results showed that T level was reduced in both female and male KC plasma, while E_2_ was increased in male KC plasma. Different levels of sex hormones are correlated with KC, which, may provide the basis of a new technique for screening and diagnosing KC with or without the assistance of current imaging techniques. Moreover, the correlations between sex hormone alterations and KC provide compelling insight into KC etiopathogenesis.

## Introduction

Keratoconus (KC) is a bilateral progressive ectatic disease with central and/or paracentral thinning and steeping of the cornea. It was first described in 1936 by Dr. Benedict Duddell. Since then, extensive research has been conducted to understand the pathophysiology of the disease (1, 2). The incidence rate is 0.05–0.23% worldwide; and the ratio of male to female incidence is 0.9–2.5:1 (3). It appears during puberty and progresses until the third or fourth decade of life (4, 5). Males develop the disease earlier and progress more rapidly than females (5).

Patients with KC always develop irregular astigmatism and myopia, leading to mild-to-marked impairment in visual acuity and quality of vision. Central or paracentral stromal thinning, rupture in the Bowman's layer, ring-shaped ferritin deposition at the basal layer of the epithelium (Fleischer's ring), fine vertical lines in the deep stroma of Descemet's membrane (Vogt's striae), and Munson's sign are typical clinical manifestations of KC (6, 7). Numerous hypotheses have been proposed, including genes, sex, eye rubbing, contact lens use, age, sun exposure, inflammation, and even pollution (2, 8–11). Recently, scientists have found that hormones, including sex hormones, prolactin, gonadotropins, thyroid hormones, glucocorticoids, and relaxin may be involved in the development of KC (8, 12–20). However, the exact mechanisms remain unclear. Many studies have shown that sex hormones cause and promote the progression of KC by influencing corneal matrix metalloproteinases (MMP) (21–23).

The present study aimed to investigate the association between sex hormones and KC, and analyze their possible mechanisms.

## Materials and Methods

This study was conducted at the Peking University Third Hospital Eye Center, Beijing, China. This study was approved by the Medical Science Research Ethics Committee of Peking University Third Hospital, and followed the tenets of the Declaration of Helsinki. Informed written consent was obtained from all subjects.

### Patient Selection

All subjects were enrolled continuously in the Peking University Third Hospital Eye Center in 2020. Patients were divided into two groups: (1) 62 eyes of 62 patients (12 females, 50 males) classified as KC and scheduled to be treated with corneal collagen cross-linking (CXL). (2) Hundred and twenty eyes of 120 patients (21 females, 99 males) with mild to moderate myopia and astigmatism were scheduled to be treated with laser vision correction (LVC). In the KC group, only the operative eye data were included. In the control group, one eye from each patient was randomly selected for comparison. All patients in the control group were examined at least twice before surgery to ensure that the patients' myopia or astigmatism was stable and conformed to the surgical indications. The diagnosis of KC was based on the presence of clinical signs (such as cornea ectasia, Fleischer's ring, Vogt's striae), central or paracentral steepening in the corneal curvature, cornea thickness, and other necessary parameters measured using topographic and tomographic maps. All diagnoses were confirmed by an experienced specialist in CXL and LVC surgery. Patients with a trend of progression in the past 12 months and that met one of the following conditions were treated with CXL: the maximum K reading increased by >1 D, mean corneal refractive power increased by >1 D, astigmatism increased by >1 D, and the spherical equivalent of manifest refraction increased by >1 D with best spectacle-corrected distance visual acuity having lost more than one line. Exclusion criteria included hormone supplementation therapy, previous ocular surgery, trauma, and systemic diseases such as immunodeficiency and desmosis.

### Examination Protocol

All subjects underwent a complete standard ophthalmological examination. The refraction errors were evaluated with a KR-8100 auto kerato-refractometer (Topcon, Tokyo, Japan), visual acuity was measured with the Standard Logarithmic Visual Acuity Chart. The anterior segment and fundus were examined with a slit-lamp microscope (YZ5F, Suzhou, China), intraocular pressure was measured with non-contact tonometers (Canon, Tokyo, Japan), and corneal parameters such as the central corneal thickness (CCT), thinnest corneal thickness (TCT), and maximum simulated keratometry (K_max_) were measured using a Pentacam (Oculus, Wetzlar, Germany) and Sirius (Costruzione Strumenti Oftalmici, Florence, Italy).

Blood samples were obtained at 10:00–13:00 from all subjects. For males, 2–3 ml blood samples were collected from the outpatient venous blood collection area 3 days before surgery, and were sent to the Laboratory of Endocrinology, Reproductive Center, Peking University Third Hospital to test the levels of sex hormones, including estriol (E_3_), estradiol (E_2_), progesterone (P), and testosterone (T) using chemiluminescence immunoassay by Immulite 2000 (Siemens, Flanders, USA). In consideration of the effect of the menstrual cycle on female sex hormones, blood samples were collected from females during their secretory phase. Once received, the levels of the four hormones are processed within approximately 24 h. For males, normal E_2_ ranges were 0.00–143.00 pmol/L and normal P ranges were 0.86–2.90 nmol/L. Male androgen had no normal ranges. For females, normal E_2_ ranges were 111.00–1007.00 pmol/L, normal P ranges were 3.00–68.00 nmol/L, and normal T ranges were 0.00–2.53 nmol/L.

### Data Analysis

Statistical analyses were performed using SPSS version 26 (SPSS, Inc., Chicago, IL, USA). The normality distribution was checked for all parameters using the Kolmogorov-Smirnov test. Age, sex hormone levels, and corneal parameters were analyzed descriptively, and the results were expressed as the mean and standard deviation for the two study groups. Because of the known sex bias for hormones, we thought analysis by sex was essential. The Mann-Whitney U test was used to evaluate differences in parameters between female and male patients. Spearman correlation analysis was used to correlate sex hormone levels, CCT, TCT, K_max_, and age. Statistical significance was set at *P* < 0.05.

## Results

**Figure 1:**
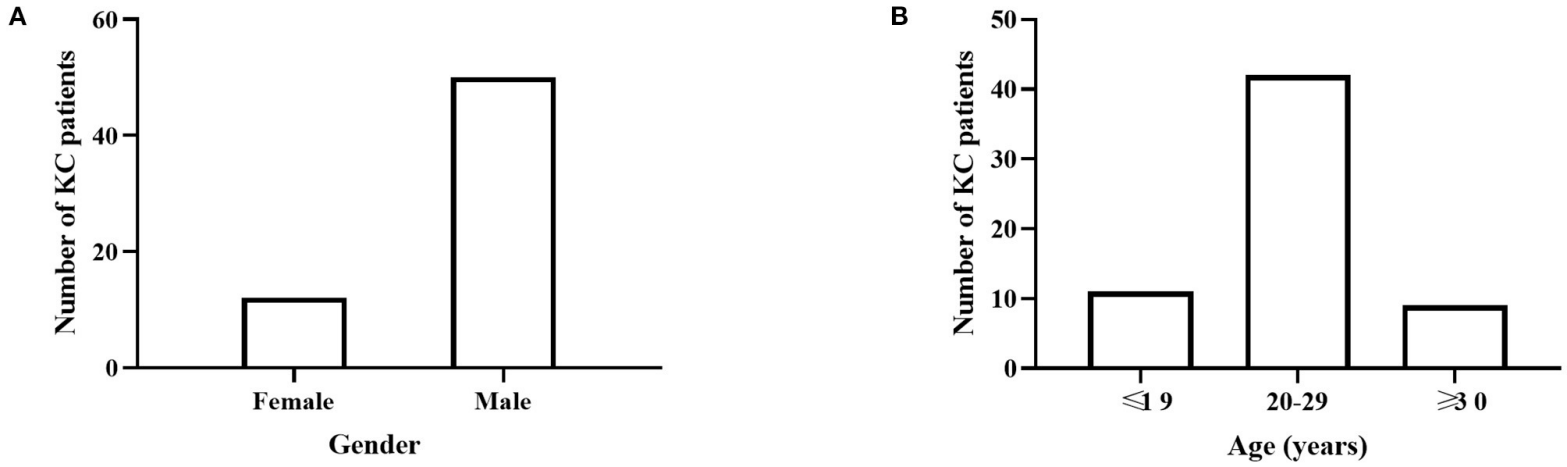
The disease distribution in different gender and age groups. **(A)** There were more males (50; 80.65%) in KC cases. **(B)** KC patients' ages were concentrated between 20 and 30 years.

Sixty-two patients with KC (12 females, 50 males) and 120 LVC patients (21 females, 99 males) were included in this study. The mean age was 23.73 ± 5.16 years for KC patients and 23.68 ± 6.10 years for LVC patients, with no significant difference between groups. Among the KC cases, there were more males (50; 80.65%), and the KC patients' ages were concentrated between 20 and 30 years (Figure 1; Table 1).

**Table 1:** Clinical and laboratory parameters.

		**KC**	**LVC**	**P value**
All	Gender (female/male)	62 (12/50)	120 (21/99)	0.740
	Age (years)	23.73 ± 5.16	23.68 ± 6.10	0.350
Female	Patients (*N*)	12	21	
	Age (years)	24.08 ± 4.42	25.05 ± 6.12	0.548
	E_3_ (nmol/L)	0.24 ± 0.00	0.25 ± 0.04	0.450
	E_2_ (pmol/L)	528.00 ± 372.86	400.27 ± 340.71	0.178
	T (nmol/L)	0.86 ± 0.33	1.18 ± 0.58	0.044
	P (nmol/L)	9.16 ± 10.13	10.92 ± 12.37	0.567
	CCT (μm)	472.67 ± 33.25	553.19 ± 39.31	<0.001
	TCT (μm)	455.17 ± 33.03	547.62 ± 39.24	<0.001
	K_max_ (D)	49.25 ± 3.23	44.46 ± 1.93	<0.001
Male	Patients (*N*)	50	99	
	Age (years)	23.64 ± 5.36	23.38 ± 6.09	0.274
	E_3_ (nmol/L)	0.24 ± 0.00	0.25 ± 0.02	0.186
	E_2_ (pmol/L)	143.75 ± 34.82	124.80 ± 43.56	0.013
	T (nmol/L)	11.59 ± 2.85	13.58 ± 4.77	0.026
	P (nmol/L)	1.44 ± 0.46	1.50 ± 0.44	0.445
	CCT (μm)	470.82 ± 35.82	553.13 ± 30.68	<0.001
	TCT (μm)	445.14 ± 39.52	548.85 ± 30.68	<0.001
	K_max_ (D)	61.89 ± 10.06	44.13 ± 1.62	<0.001

*LVC, laser vision correction; KC, keratoconus; E3, estriol; E2, estradiol; P, progesterone; T, testosterone; CCT, central corneal thickness; TCT, thinnest corneal thickness; K_max_, maximum simulated keratometry. All data are expressed as mean ± SD*.

Table 1 shows the demographic, morphological, and laboratory parameters of the female KC and LVC groups. From the data above, we could find that the concentration of T in the KC group was significantly lower than the LVC group (0.86 ± 0.33 vs. 1.18 ± 0.58 nmol/L; *P* = 0.044). We performed Pentacam and Sirius examinations, and CCT, TCT, and K_max_ were recorded for each patient. The mean CCT, TCT, and K_max_ values differed significantly between the groups (*P* < 001). In the male KC group, E_2_ was higher than in the LVC group (143.75 ± 34.82 vs. 124.80 ± 43.56 pmol/L; *P* = 0.013), while T was significantly lower (11.59 ± 2.85 vs. 13.58 ± 4.77 nmol/L; *P* = 0.026). However, there was no difference between the KC and LVC groups in terms of E_3_ and P (*P* = 0.186, *P* = 0.445).

**Figure 2:**
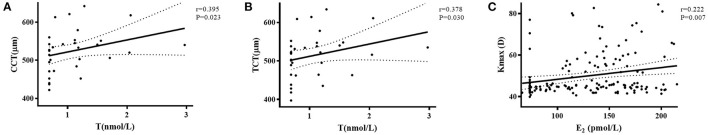
The correlation of plasma sex hormone levels to CCT, TCT, and K_max_ in female and male groups. **(A)** The level of T positively correlated with CCT (*r* = 0.395, *P* = 0.023) in female patients. **(B)** The level of T positively correlated with TCT (*r* = 0.378, *P* = 0.030) in female patients. **(C)** The level of E_2_ positively correlated with K_max_ (*r* = 0.222, *P* = 0.007) in male patients.

Figure 2 shows that the level of T positively correlated with CCT (*r* = 0.395, *P* = 0.023) and TCT (*r* = 0.378, *P* = 0.030) in female patients. A positive correlation was found between E_2_ and K_max_ (*r* = 0.222, *P* = 0.007) in male patients. E_3_ and P were not correlated with any of the KC parameters.

## Discussion

With the progression of science and technology, the early diagnosis and treatment of KC are more and increasingly accurate and cutting-edge, but the etiopathogenesis of this important kind of corneal ectasia disease has not been completely investigated. Exploring the etiopathogenesis of KCs is difficult, but essential. Hypotheses have proposed that genetic, immunological, metabolic, and endocrinological factors may be involved in the progression of KC. Furthermore, many reports have shown a significant relationship between sex hormones and KC. Some case reports have shown increased tendency for corneal ectasia in pregnancy and hormone replacement therapies and reveal corneal graft rejection in pregnancy. Yuksel et al. found that females treated with *in-vitro* fertilization always have progression in KC (24). In Emilio's report, a 49-year-old woman with previously stable KC had got late-onset KC progression when starting treatment for endometriosis using an estrogenic activity regulator with tibolone (12). Spoerl et al. exposed porcine corneas to 10 μmol/L of β-estradiol for 7 days, and found that the corneal thickness increased, but the biomechanics decreased (25). Indeed, studies have reported that sex hormones and the expression of matrix metalloproteinase-9 (MMP-9) and MMP-2 in the corneal epithelium, stroma, and tear fluid of patients with KC may be associated with the severity of KC. Taken together, the fluctuating levels of MMP-2, MMP-9 in corneas with KC and tear fluid may be regulated by sex hormones, and may additively contribute to the progression of corneal ectasia.

Plasma concentration screening, clinical observations, and clinical examinations were used to determine the correlation between KC and sex hormones. It was thought that increased levels of sex hormones may be associated with the etiopathogenesis and progression of KC due to some mechanism of action. First, c-fos, an estrogen-target gene, is involved in cell proliferation, differentiation, apoptosis, oncogenesis, and invasion. Pan found that both c-fos and MMP-9 protein levels were higher in females with endometriosis than in those without endometriosis. A positive correlation between c-fos and MMP-9 protein levels was found, which suggests that c-fos may be regulated by sex hormones and promote the gene expression of MMP-9 in the development of endometriosis (26). Therefore, estrogen may induce the expression of MMP-9 with KC in a similar way. Second, inflammatory cytokines have been reported as key factors in the pathogenesis of KC, which are stimulated by sex hormone receptors and then stimulate MMP genes in the human cornea (27). Sex hormone receptor mRNA exists in various ocular tissues, including the cornea. Ayan et al. found that the expression of progesterone and androgen receptors in the corneal epithelium was higher in patients with KC by using quantitative polymerase chain reaction (qPCR) (28). Suzuki et al. studied the expression of proinflammatory cytokines, estrogen, and MMP genes in the human cornea, and found that 17β-estradiol could stimulate the expression of various proinflammatory cytokines, such as IL-6, IL-1β, IL-8, and GM-CSF, and stimulate MMP genes in immortalized human corneal epithelial cells (29, 30). T level regulates some systemic factors, such as IL-16 and stem cell factors which may affect the corneal microenvironment (31). There is a direct link between enhanced proinflammatory cytokines and thinner corneas in patients with KC, and the phenomenon of increased curvature and enhanced inflammatory factors shows that inflammatory factors may contribute to disease severity. Activated MMP-9 and−2 can degrade the basement membrane, lead to cytokine release, and regulate proinflammatory cytokines.

Although the plasma levels of sex hormones were approximately in the normal range in patients with KC and LVC, the plasma levels of E_2_ and T showed a statistically difference, suggesting that even in the normal range, the sex hormone might have certain influences on the cornea.

This study has several limitations. The present study had a cross-sectional design, and the findings must be confirmed via longitudinal studies that require larger patient populations. Sex hormones were not measured in the tear fluid. In addition, there are changes in many other hormones, such as growth hormone, around the age of 25. Thus, further research is required that examines their role in KC.

In conclusion, although the specific mechanism between sex hormones and KC appears not to have been investigated, it is proposed that sex hormones may play an essential role in the development of KC. The results of our study might be helpful as a basis for further studies to elucidate the etiopathogenesis of KC and explore the role of sex hormones in the treatment of KC in the future.

## Data Availability Statement

The raw data supporting the conclusions of this article will be made available by the authors, without undue reservation.

## Ethics Statement

The studies involving human participants were reviewed and approved by The Medical Science Research Ethics Committee of Peking University Third Hospital. Written informed consent to participate in this study was provided by the participants or their legal guardian/next of kin.

## Author Contributions

XZ, YY, and TS were responsible for the initial plan, study design, data collection, data extraction, data interpretation, manuscript drafting, statistical analysis, and conducting the study. YZ and YC were responsible for data collection, extraction, and critical revisions of the manuscript. YC was the guarantor for this article and has full responsibility for this study. All authors contributed to the article and approved the submitted version.

## Funding

This study was supported by grant of Key Clinical Innovation Program of Peking University Third Hospital, Category A, No. Y65495–05.

## Conflict of Interest

The authors declare that the research was conducted in the absence of any commercial or financial relationships that could be construed as a potential conflict of interest.

## Publisher's Note

All claims expressed in this article are solely those of the authors and do not necessarily represent those of their affiliated organizations, or those of the publisher, the editors and the reviewers. Any product that may be evaluated in this article, or claim that may be made by its manufacturer, is not guaranteed or endorsed by the publisher.
